# Intimate partner violence and its association with skilled birth attendance among women in Nigeria: evidence from the Nigeria Demographic and Health Surveys

**DOI:** 10.1186/s12884-022-04989-1

**Published:** 2022-08-30

**Authors:** Chukwuechefulam Kingsley Imo, Nnebechukwu Henry Ugwu, Ukoji Vitalis Ukoji, Uche Charlie Isiugo-Abanihe

**Affiliations:** 1 Department of Sociology, Faculty of the Social Sciences, Adekunle Ajasin University, Akoko-Akungba, Ondo State Nigeria; 2 Demography and Population Studies Programme, Schools of Public Health and Social Sciences, University of the Witwatersrand, Johannesburg, South Africa; 3 Institute for Development Studies, University of Nigeria, Enugu Campus, Nsukka, Nigeria; 4 Department of Sociology, Faculty of Social and Management Sciences, Nigeria Police Academy, Kano, Nigeria; 5 Department of Sociology, University of Ibadan, Ibadan, Oyo State Nigeria

**Keywords:** Skilled birth attendance, Intimate partner violence, Childbearing women, Trends, Nigeria

## Abstract

**Background:**

Intimate Partner Violence (IPV) has been identified as a violation of human rights and a major public health challenge. IPV against women has negative effects on women’s mental well-being and leads to unfavourable health outcomes through poor maternal healthcare services utilisation, especially skilled birth attendance (SBA). This study examined the trends in IPV and SBA, as well as the different forms of IPV as predictors of SBA in Nigeria.

**Methods:**

Data for the study were derived from a nationally representative weighted sample of 34,294 women selected and interviewed for the questions on the domestic violence module in the three consecutive Nigeria Demographic and Health Surveys conducted in 2008, 2013 and 2018. Descriptive and analytical analyses were carried out, including frequency distribution and binary logistic regression model at the multivariate level. The results of the explanatory variables were expressed as odds ratio (OR) and 95% confidence intervals (CI).

**Results:**

The prevalence of emotional and physical IPV among the sampled women decreased in 2013 from 2008 but later increased in 2018. Sexual IPV increased from 4.1% in 2008 to 7.6% in 2018, while births delivered with the assistance of skilled providers increased from 37.7% in 2008 to 50.8% in 2018. The likelihood of using SBA significantly decreased among women who experienced emotional IPV in 2008 (aOR: 0.74; CI: 0.63–0.87) and sexual IPV in 2018 (aOR: 0.62; CI: 0.45–0.86). Women who experienced physical IPV were more likely to use SBA in 2008, 2013 and 2018 (aOR: 1.72; CI: 1.55–1.92; aOR: 1.40; CI: 1.26–1.56 and aOR: 1.33; CI: 1.15–1.54, respectively). The covariates have varying degrees of influence on SBA across the survey years.

**Conclusions:**

The showed that the prevalence of emotional and physical IPV increased in 2018 after a decrease in 2013, with an increase in sexual IPV and the use of SBA across the survey years. Also, emotional and sexual IPV, unlike physical IPV are associated with low chances of using SBA. There is a need for more pragmatic intervention programmes towards eliminating all forms of violence against all women, reducing maternal and child mortality and promoting the empowerment of women.

## Background

Maternal healthcare, especially among women of reproductive age, is an important part of the public health system [1]. Apart from being a major indicator in measuring the level of healthcare performance, delivery system and developmental indices, proper care during pregnancy and delivery are important for the well-being of both the mother and child [2]. Globally, preventable causes related to pregnancy and childbirth lead to the deaths of many women and children, with the vast majority of these deaths occurring in low-resource settings [3]. One of the reasons for most maternal and child deaths in low- and middle-income countries, of which the majority have been recorded in sub-Saharan Africa (SSA) where Nigeria is dominant, is the lack of skilled birth attendance (SBA) during delivery [4]. To save the lives of women and newborns, the World Health Organisation (WHO) advocates for high-quality maternal and newborn care in health facilities through proper antenatal care (ANC) visits during pregnancy necessitating skilled care at every birth [5].

Although, it has been documented that the degree of progress recorded in skilled birth attendance (SBA) during delivery is not commensurate with an increase in ANC utilization [6, 7]. The presence of a skilled health professional, such as a doctor, nurse or midwife who is trained to manage pregnancies, childbirth and the immediate postnatal period is one of the central elements in reducing maternal and child deaths [8–10]. Previous studies have shown that socioeconomic, cultural and other related factors influence SBA utilisation [11–14]. In addition, cultural and institutional norms like male-headed households and gender-based power inequalities, through gender-based violence adversely affect women’s sexual and reproductive health [15, 16]. The roles of psychosocial barriers in accessing reproductive healthcare services in developing countries, as well as the prevalence of intimate partner violence in many contexts have been documented [17].

Intimate Partner Violence (IPV) is one of the most common forms of violence against women and includes physical, sexual, and emotional abuse and controlling behaviours by a partner; it has been identified as a major public health challenge [18, 19]. Women experience IPV more than their men counterparts worldwide, as well as in SSA [20, 21]. Globally, WHO estimated that from 2000 to 2018 across 161 countries and areas, about 1 in 3 (30%) women has been subjected to some form of IPV or non-partner violence in their lifetime [22]. In Nigeria, the prevalence of all forms of spousal violence (physical, sexual or emotional) increased from 31% in 2008 and 25% in 2013 to 36% in 2018 [10, 23, 24]. Previous studies have shown that IPV violates human rights and adversely affects economic and social development [21, 25, 26]. Consequently, women who are victims of IPV might experience negative health outcomes, including a lack of utilisation of SBA through its negative impact on women’s mental well-being [27]. In his 2006 declaration, the former United Nations Secretary-General, Ban Ki-Moon stated that violence against women is never acceptable, excusable and tolerable [28]. Nevertheless, IPV against women persists in sub-Saharan Africa, which Nigeria is part of [20, 29]. This is irrespective of the administration of gender-based policies and laws geared towards reducing violence against women and improving women’s health in Nigeria [30, 31]. For instance, one in three women in Nigeria reported having ever experienced IPV [23]. There have been several studies on the prevalence of IPV and identified linkage with the use of maternal healthcare services [29, 32–36]. It is, therefore, evident that there is a paucity of empirical research on the trends in IPV and SBA, as well as the relationship between IPV and SBA in Nigeria.

Understanding the trends in IPV and SBA, as well as the independent influence of different forms of IPV and SBA, is essential for the design and assessment of interventions to improve women’s sexual and reproductive health, as well as achieve sustainable development goals (SDGs) targets. These include reducing maternal and child mortality, eliminating all forms of violence against all women and promoting the empowerment of women. This study examined the trends in IPV and SBA, and the different forms of IPV as predictors of SBA in Nigeria using three consecutive NDHS. The reason is to investigate the achievement so far in reducing IPV against women and its influence on the use of SBA which affects women and children’s health outcomes years after the UN declaration on violence against women and the administration of gender-based policies and laws in Nigeria.

## Methods

### Data source and sample

The data for this study were obtained from the three consecutive Nigeria Demographic and Health Surveys (NDHS) datasets conducted in 2008, 2013 and 2018. The surveys are parts of the Demographic and Health Surveys (DHS) programme which ICF provided technical assistance targeting women aged 15–49 years and men aged 15–59 years in randomly selected households across Nigeria. The surveys provided information on 33,385 (2008 NDHS); 38,948 (2013 NDHS) and 41,821 (2018 NDHS) women sampled across Nigeria. Each of the surveys as part of the DHS programme collected nationally representative data on domestic violence, especially IPV, births and skilled attendance from women aged 15–49 years. Detailed reports of the adopted methods and procedures in the collection of data for the surveys can be found in the final reports [10, 23, 24]. For this study, all the women who were not selected and interviewed for the questions on the domestic violence module, as well as those who reported not being married and living with partners, and did not give birth in the five years before the surveys were excluded from the analyses. Three datasets were combined giving a total of 34,294 currently married and cohabiting women who had given birth at least to a child in the five years before the surveys. The women were selected from nationally representative samples of 13,298 from 2008 NDHS; 14,633 from 2013 NDHS; and 6,363 from 2018 NDHS.

### Outcome variable – skilled birth attendance

The outcome variable was measured by asking the respondents about assistance during delivery. Respondents whose deliveries were assisted by a doctor, nurse/midwife, or auxiliary nurse/midwife were categorised as having ‘skilled birth attendance’ – coded ‘1’, while deliveries assisted by other community health extension workers, traditional birth attendants, friends/relatives or no assistance were defined as ‘unskilled birth attendance’ – coded ‘0’. The classification of the outcome variable was based on previous evidence [10, 23, 24].

### Explanatory variables – Intimate Partner Violence (IPV)

The explanatory variables comprised the three measures of violence derived from asking women to respond ‘yes’ or ‘no’ to questions on if their husbands/partners ever abused them physically, sexually and emotionally. This study adopted the ‘generate’ and ‘replace’ commands of the Stata and each type of partner violence was aggregated into a single variable which reflects each of the three forms of intimate partner IPV (physical, sexual, and emotional) against women. Therefore, women who responded in the affirmative to any of the questions which constitute evidence of IPV were coded ‘1’, while otherwise coded ‘2’. The descriptions of the three measures are listed below:

- *Physical violence*: Partner pushed, shook or threw something at you; slapped; twisted your arm or pulled your hair; punched you with his fist or with something that could hurt you; kicked, dragged or beat you up; tried to choke you or burn you on purpose; threatened or attacked you with a knife, gun, or any other weapon.
- *Sexual violence*: Partner physically forced you, especially with threats, to have sexual intercourse with him even when you did not want to.
- *Emotional violence*: Partner said or did something to humiliate you in front of others, threatened to hurt or harm you, insulted you or made you feel bad about yourself.

The covariates included in the analysis were age, marital status, educational attainment, employment status, wealth quintile, place of residence, health insurance coverage, decision-making autonomy on healthcare and earnings. Some variables were re-categorised to make interpretation simpler and more meaningful. For instance, the current age of the women: 15–24/25–34/35 + years; Educational attainment: no education/ primary/secondary or tertiary; and wealth quintile: lowest/middle/highest; as well as decision-making autonomy on respondent’s healthcare and how to spend her earnings: husband/partner alone/jointly or alone. Women’s decision-making autonomy in this context is defined as the extent of their independence on matters about their health and how to spend their earnings without having to obtain permission; it is vital for maternal healthcare utilisation [37]. The documented significant association with sexual practices in the literature and their availability in the dataset guided the selection of all the variables.

### Statistical analysis

The datasets were carefully checked for missing values which were excluded and weighted with the appropriate sampling weights as per the Demographic and Health Survey (DHS) sampling scheme using Stata software (version 15.1). Three levels of analyses (univariate, bivariate and multivariate) were employed. At the univariate level, descriptive statistics related to the characteristics of the study population, forms of IPV and use of SBA were generated through frequency and percentage. Using the Pearson chi-square test, unadjusted binary logistic regression analyses were adopted at the bivariate level to investigate the independent effect of each explanatory variable on the outcome variable. At the multivariate level, binary logistic regression models were used to measure the odds ratio (OR) of the association between skilled attendance at delivery and the explanatory variables. The unadjusted and adjusted binary logistic regression analyses of the association between intimate partner violence and the use of skilled birth attendance were carried out at the multivariate level. Possible associations between each of the explanatory variables and the outcome variable were assessed by observing the *p*-values [38]. The results of the explanatory variables were expressed as OR with 95% confidence intervals (CI). Therefore, an explanatory variable with OR greater than 1.00 implied an increased likelihood of the outcome, while it is the opposite when the OR is less than 1.00 [39].

## Results

### Description of the study population

The weighted descriptive statistics of the sampled women as presented in Table 1 showed that more than 48% of the women were aged 25–34 years across the three survey years. Over the 10 years, the proportion of women with no formal education decreased from 48.2% in 2008 to 37% in 2018, while those with secondary/tertiary education increased from 29.4% in 2008 to 46.9% in 2018. The percentage of women who were currently working slightly increased from 64.8% in 2008 to 69.1% in 2018. The results further showed that the largest proportions of the women were found in the lowest wealth quintile households with a decrease from 48.9% in 2008 to 39.8% in 2018. There was a steady decrease in the percentage of women residing in rural areas. An overwhelming majority of the women were not covered by health insurance across the survey years. Concerning decision-making on women’s healthcare and earnings, the result showed that most decisions on women’s healthcare were made by their husbands/partners, while over 65% of the women enjoyed decision-making autonomy on how their earnings are spent across the survey years.

**Table 1:** Percent distribution of respondents by background characteristics

Variable/category	2008 NDHS	2013 NDHS	2018 NDHS
	Number (%)	Number (%)	Number (%)
**Age**			
15 – 24	3,575(26.8)	3,738(25.6)	1,440(22.6)
25 – 34	6,457(48.6)	7,191(49.1)	3,172(49.9)
35 and above	3,266(24.6)	3,704(25.3)	1,751(27.5)
**Educational attainment**			
No education	6,405(48.2)	6,406(43.8)	2,355(37.0)
Primary	2,977(22.4)	2.948(20.2)	1,023(16.1)
Secondary/tertiary	3,916(29.4)	5,279(36.0)	2,985(46.9)
**Employment status**			
Not working	4,651(35.2)	4,523(30.9)	1,913(30.1)
Currently working	8,568(64.8)	10,110(69.1)	4,450(69.9)
**Wealth quintile**			
Lowest	6,497(48.9)	6,302(43.1)	2,531(39.8)
Middle	2,508(18.9)	2,817(19.2)	1,369(21.5)
Highest	4,293(32.2)	5,514(37.7)	2,463(38.7)
**Place of residence**			
Urban	3,732(28.1)	5,188(35.5)	2,565(40.3)
Rural	9,566(71.9)	9,445(64.5)	3,798(59.7)
**Covered by health insurance**			
No	13.056(98.5)	14.349(98.1)	6,203(97.6)
Yes	195(1.5)	284(1.9)	155(2.4)
**Decision on respondent's health**			
Husband/partner alone	7,684(57.9)	8,872(60.6)	3,439(54.1)
Respondent and partner	4,556(34.3)	4,908(33.5)	2,327(36.6)
Respondent alone	1,034(7.8)	853(5.8)	597(9.4)
**Decision on how to spend respondent's Earnings**			
Husband/partner alone	1.088(15.2)	1,007(10.6)	377(9.6)
Respondent and partner	1,381(19.3)	1,879(19.8)	939(23.9)
Respondent alone	4,684(65.5)	6,586(69.6)	2,615(66.5)

### Trends in intimate partner violence and skilled birth attendance

**Fig. 1:**
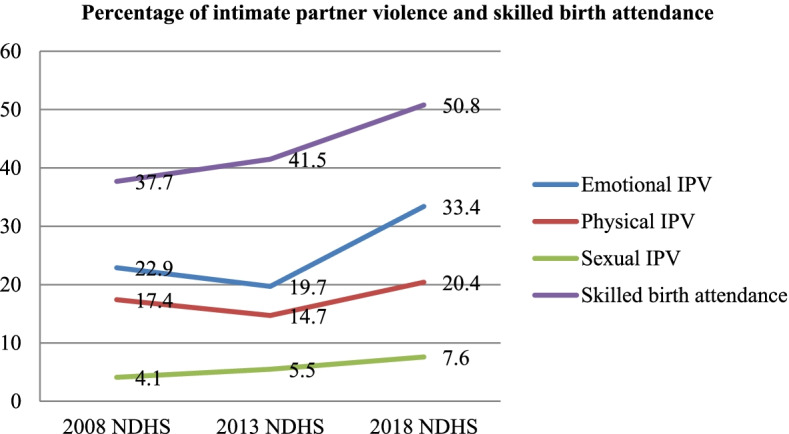
Trends in intimate partner violence and skilled birth attendance by survey years (%)

Figure 1 showed the trends in intimate partner violence and skilled birth attendance among the sampled women for this study between 2008 and 2018. Over the 10 years, after a slight decrease in emotional IPV from 22.9% in 2008 to 19.7% in 2013, it increased to 33.4% in 2018. A similar trend was observed for Physical IPV which decreased from 17.4% in 2008 to 14.7% in 2013 but later increased to 20.4% in 2018. The results showed a gradual increase in sexual IPV from 4.1% in 2008 to 7.6% in 2018. Births delivered with the assistance of skilled providers continuously increased from 37.7% in 2008 to 50.8% in 2018.

### Bivariate association between skilled birth attendance and all the explanatory variables

The unadjusted binary logistic regression was adopted at the bivariate level to investigate the independent effect of each explanatory variable with the outcome variable in Table 2. The results of the explanatory variables expressed as OR with 95% CI indicated that variable with OR greater than 1.00 implied an increased likelihood of the outcome, while it is the opposite when the OR is less than 1.00. The results showed that all the explanatory variables were significantly associated with SBA across the survey years, except for physical IPV in 2018 and sexual IPV in 2008. The likelihood of using SBA significantly reduced among women who experienced emotional IPV in 2008 (OR: 0.81; CI: 0.74–0.88) and 2018 (OR: 0.83; CI: 0.75–0.92) but increased in 2013 (OR: 1.34; CI: 1.23–1.45). Experiencing physical IPV among the sampled women was significantly associated with an increase in the use of SBA in 2008 and 2013 (OR: 1.41; CI: 1.28–1.54 and OR: 1.43; CI: 1.31–1.57, respectively). Also, the likelihood of using SBA was significantly reduced among women who experienced sexual IPV in 2013 and 2018 (OR: 0.78; CI: 0.67–0.90 and OR: 0.60; CI: 0.49–0.72, respectively).

**Table 2:** Unadjusted bivariate analysis of the association between the explanatory variables and skilled birth attendance by survey years

Characteristics	2008 NDHS	2013 NDHS	2018 NDHS
	**OR(95% CI)**	**OR(95% CI)**	**OR(95% CI)**
**Emotional IPV**			
No (Ref.)	1.00	1.00	1.00
Yes	0.81(0.74–0.88)***	1.34(1.23–1.45)***	0.83(0.75–0.92)***
**Physical IPV**			
No (Ref.)	1.00	1.00	1.00
Yes	1.41(1.28–1.54)***	1.43(1.31–1.57)***	1.03(0.91–1.16)
**Sexual IPV**			
No (Ref.)	1.00	1.00	1.00
Yes	1.10(0.92–1.31)	0.78(0.67–0.90)**	0.60(0.49–0.72)***
**Age**			
15 – 24 (Ref.)	1.00	1.00	1.00
25 – 34	1.80(1.64–1.96)***	1.74(1.60–1.89)***	1.77(1.56–2.01)***
35 and above	1.43(1.29–1.58)***	1.63(1.48–1.79)***	1.62(1.41–1.87)***
**Educational attainment**			
No education (Ref.)	1.00	1.00	1.00
Primary	5.42(4.89–6.02)***	5.28(4.77–5.86)***	5.39(4.57–6.35)***
Secondary/tertiary	22.13(19.93–24.58)***	19.87(18.06–21.87)**	18.24(15.87–20.98)***
**Employment status**			
Not working (Ref.)	1.00	1.00	1.00
Currently working	2.06(1.91–2.23)***	1.83(1.70–1.97)***	2.24(2.01–2.50)***
**Wealth quintile**			
Lowest (Ref.)	1.00	1.00	1.00
Middle	3.84(3.44–4.28)***	4.79(4.31–5.33)***	4.21(3.65–4.86)***
Highest	19.61(17.74–21.66)***	20.67(18.78–22.76)***	14.51(12.66–16.64)***
**Place of residence**			
Urban (Ref.)	1.00	1.00	1.00
Rural	0.19(0.17–0.21)***	0.14(0.13–0.15)***	0.20(0.18–0.22)***
**Covered by health insurance**			
No (Ref.)	1.00	1.00	1.00
Yes	15.79(9.83–25.39)***	10.42(7.31–14.87)***	4.15(2.78–6.21)***
**Decision on respondent's health**			
Husband/partner alone (Ref.)	1.00	1.00	1.00
Respondent and partner	2.77(2.57–2.99)***	3.68(3.42–3.96)***	3.65(3.26–4.08)***
Respondent alone	3.77(3.30–4.31)***	4.97(4.26–5.77)***	3.09(2.58–3.70)***
**Decision on how to spend respondent’s earnings**			
Husband/partner alone (Ref.)	1.00	1.00	1.00
Respondent and partner	2.50(2.12–2.94)***	1.46(1.25–1.70)***	2.87(2.23–3.68)***
Respondent alone	0.87(0.76–1.00)*	0.71(0.62–0.81)***	1.11(0.89–1.37)

*IPV* Intimate partner violence, *Ref.* Reference category

^*^p < 0.05; **p < 0.01; ***p < 0.001

Concerning the covariates, the results showed that the likelihood of using SBA significantly increased among older women aged 25 years and above relative to those aged 15–24 years across the survey years. The results further showed significantly strong effects of having higher education, being employed and an increase in household wealth quintile on the use of SBA among the sampled women across the survey years. Also, 81%, 86%, and 80% of births among rural women were significantly less attended by a skilled birth attendant, compared to their urban counterparts, in 2008, 2013, and 2018, respectively. The likelihood of using SBA significantly increased among women who reported being covered by health insurance across the survey years. The results in Table 2 further showed that women participating in decision-making and enjoying autonomy in their healthcare significantly increased the likelihood of using SBA across the survey years. Similar significantly increased results of using SBA were observed among women, who participated in decision-making on how to spend their earnings with partners in 2008, 2013 and 2018. Surprisingly, the chances of using SBA significantly reduced among women who enjoyed decision-making autonomy on how to spend their earnings in 2008 (OR: 0.87; CI: 0.76–1.00) and 2013 (OR: 0.71; CI: 0.62–0.81) compared with those whose partners independently made decisions on their earnings.

### Multivariate analysis of the association between the explanatory variables and skilled birth attendance by survey years

The results of the adjusted associations between the explanatory variables and SBA are presented in Table 3. Two models (Models 1 and 2) were fitted for the outcome variable. Model 1 adjusted for the main explanatory variables, while Model 2 adjusted for the main explanatory variables and covariates to examine their simultaneous influence on SBA. The results in Model 1 showed that the likelihood of using SBA significantly decreased among women who experienced emotional IPV in 2008 and 2018 (aOR: 0.66; CI: 0.60–0.72 and aOR: 0.80; CI: 0.71–1.09, respectively), but increased in 2013 (aOR: 1.29; CI: 1.77–1.42). The chance of using SBA significantly increased among women who experienced physical IPV across the survey years. The results further showed that women experiencing sexual IPV were associated with a decreased likelihood of using SBA, though significant in 2013 and 2018 (aOR: 0.57; CI: 0.48–0.67 and aOR: 0.57; CI: 0.47–0.70, respectively) compared with their counterparts in the reference category.

**Table 3:** Adjusted multivariate analysis of the association between the explanatory variables and skilled birth attendance by survey years

Characteristics	2008 NDHS		2013 NDHS		2018 NDHS	
	Model 1	Model 2	Model 1	Model 2	Model 1	Model 2
	**aOR(95% CI)**	**aOR(95% CI)**	**aOR(95% CI)**	**aOR(95% CI)**	**aOR(95% CI)**	**aOR(95% CI)**
**Emotional IPV**						
No (Ref.)	1.00	1.00	1.00	1.00	1.00	1.00
Yes	0.66(0.60–0.72)***	0.74(0.63–0.87)***	1.29(1.17–1.42)***	1.11(0.96–1.29)	0.80(0.71–0.91)**	0.90(0.74–1.09)
**Physical IPV**						
No (Ref.)	1.00	1.00	1.00	1.00	1.00	1.00
Yes	1.72(1.55–1.92)***	1.12(0.94–1.35)	1.40(1.26–1.56)***	0.91(0.78–1.08)	1.33(1.15–1.54)***	1.03(0.82–1.29)
**Sexual IPV**						
No (Ref.)	1.00	1.00	1.00	1.00	1.00	1.00
Yes	0.97(0.80–1.17)	0.98(0.72–1.34)	0.57(0.48–0.67)***	0.91(0.71–1.17)	0.57(0.47–0.70)***	0.62(0.45–0.86)**
**Age**						
15 – 24 (Ref.)		1.00		1.00		1.00
25 – 34		1.31(1.11–1.54)**		1.20(1.04–1.38)*		1.19(0.95–1.48)
35 and above		1.36(1.13–1.63)**		1.47(1.26–1.72)***		1.26(0.99–1.59)
**Educational attainment**						
No education (Ref.)		1.00		1.00		1.00
Primary		3.06(2.62–3.59)***		2.84(2.46–3.29)***		3.25(2.59–4.09)***
Secondary/tertiary		7.14(6.02–8.46)***		5.86(5.06–6.80)***		6.63(5.33–8.24)***
**Employment status**						
Not working (Ref.)		1.00		1.00		1.00
Currently working		0.99(0.74–1.34)		0.94(0.67–1.32)		1.03(0.68–1.57)
**Wealth quintile**						
Lowest (Ref.)		1.00		1.00		1.00
Middle		1.96(1.66–2.32)***		2.27(1.96–2.63)***		1.93(1.57–3.38)***
Highest		4.76(3.99–5.66)***		4.39(3.76–5.14)***		3.28(2.63–4.09)***
**Place of Residence**						
Urban (Ref.)		1.00		1.00		1.00
Rural		0.65(0.56–0.76)***		0.44(0.39–0.50)***		0.57(0.48–0.68)***
**Covered by health insurance**						
No (Ref.)		1.00		1.00		1.00
Yes		2.28(1.15–4.50)*		2.21(1.40–3.49)**		1.68(0.89–3.14)
**Decision on respondent's health**						
Husband/partner alone (Ref.)		1.00		1.00		1.00
Respondent and partner		1.50(1.30–1.74)***		1.63(1.45–1.85)***		1.38(1.15–1.67)**
Respondent alone		2.28(1.84–2.83)***		1.94(1.58–2.38)***		1.63(1.24–2.13)***
**Decision on how to spend respondent's earnings**						
Husband/partner alone (Ref.)		1.00		1.00		1.00
Respondent and partner		1.25(1.00–1.56)		0.69(0.57–0.85)***		1.31(0.95–1.79)
Respondent alone		0.75(0.63–0.89)**		0.58(0.49–0.69)***		0.96(0.74–1.26)

*IPV* Intimate partner violence, *Ref.* Reference category

^*^p < 0.05; **p < 0.01; ***p < 0.001

After adjusting for the main explanatory variables and covariates in Model 2, the results showed that the likelihood of using SBA significantly decreased among women who reported having experienced emotional IPV in 2008 (aOR: 0.74; CI: 0.63–0.87) in comparison with those who were not emotionally abused by partners. A similar result was observed among women who experienced sexual IPV in 2018. The likelihood of using SBA significantly increased among older women aged 25 years compared with those aged 15–24 years in 2008 and 2013. The results further showed that the chance of using SBA significantly increased with an increase in women’s education and household wealth quintile, compared with those in the reference categories across the survey years. Concerning the place of residence, 35%, 56%, and 43% of rural women were significantly less attended by a skilled birth attendant, compared to those residing in the urban areas in 2008, 2013, and 2018, respectively. The likelihood of using SBA increased across the survey years, but significantly increased 2.28 times (95% CI: 1.15–4.50) in 2008 and 2.21 times (95% CI: 1.40–3.49) in 2013. The results further showed that women participating and enjoying decision-making autonomy on their healthcare significantly their chances of using SBA in 2008, 2013 and 2018. Surprisingly, the likelihood of suing SBA significantly decreased among women who reported having decision-making autonomy on how to spend their earnings in 2008 and 2013 (aOR: 0.75; CI: 0.63–0.89 and aOR: 0.58; CI: 0.49–0.69, respectively) compared with those whose partners made independent decisions on their earnings.

### Discussion of findings

This empirical study reporting the trends in IPV and SBA, and the influence of different forms of IPV on SBA among women who were selected and interviewed for the questions on the domestic violence module of the three consecutive NDHS reports marks a departure from previous studies in Nigeria. The women included in the analyses were those who reported being married and living with partners and had given birth in the five years before the surveys. Through this study, our findings revealed trends in IPV and SBA and identified different forms of IPV predicting SBA over the survey years.

Overall, our findings showed that the prevalence of emotional and physical IPV among the sampled women decreased in 2013 from 2008, but later increased in 2018. Also, the pattern of sexual IPV is lower than emotional and physical IPV with a gradual increase across the survey years. The findings on the low prevalence of sexual IPV might be related to the fact that most sexually victimized women are not likely to report such attacks for fear of discrimination and feeling shame [40]. Our findings further showed that births delivered with the assistance of skilled providers continuously increased across the survey years. Despite the upward trend in the use of SBA, our findings that births assisted by skilled providers are low considering pragmatic policies towards access to quality healthcare corroborate the observations of previous studies in Nigeria [41] and Ghana [42]. However, the plausible explanations for the upward trend in the use of SBA among women could be attributed to the government policy which encouraged the recruitment of skilled personnel [10] and the autonomy granted to the states and Local Government Authorities in the healthcare system for planning and delivery of health services in Nigeria [43].

The results of the adjusted measures of IPV showed that women experiencing different forms of IPV have varying degrees of influence on their chances of using SBA. For instance, our findings showed that the chance of using SBA significantly decreased among women who experienced emotional IPV in 2008 and 2018 but increased in 2013. Also, women experiencing sexual IPV were associated with less likelihood of using SBA across the survey years, though significant in 2013 and 2018. Our findings on the negative influence of emotional and sexual IPV on the chance of using SBA corroborate the observations of previous studies in Nigeria [29, 35] and other countries [44–46]. This has some policy implications for achieving SDGs since women who are emotionally and sexually abused might experience feelings of confusion, anxiety, shame, aversion and powerlessness which adversely influence their sexual and reproductive healthcare decisions, especially consulting a skilled birth attendant during delivery [40, 47]. The implication is that emotional and sexual violence may play less noticeable but important roles in the poor utilisation of maternal healthcare services, especially the use of SBA during delivery. In contrary with the observation of a previous study that experiencing physical violence was negatively associated with the use of skilled assistance during delivery [48], the findings of this study a significantly positive relationship between physical IPV and the chance of using SBA among women in Models 1 and 2, Table 3 across the survey years. On the other hand, the findings are consistent with the observations of previous studies in Nigeria and other sub-Saharan African countries that women who experienced physical violence were more likely to use SBA [35, 44, 48, 49]. This plausibly could be attributed to the fact that women who are physically abused by their partners tend to visit skilled healthcare providers, especially during pregnancy for emergency treatment and management of injuries sustained to avoid the likelihood of miscarriage, stillbirth or pre-term delivery.

Consistent with a study in Bangladesh [45], our findings revealed that women becoming older were significantly associated with the use of SBA. The possible explanation might be attributed to the fact that as women grow older, they become generally more experienced and knowledgeable, even in the face of IPV to make positive healthcare decisions, including seeking skilled healthcare services. This is in contrast with the observation of a study conducted in Ethiopia that older women consider seeking healthcare services, irrespective of the provider because of their previous childbirth experiences [50]. In corroboration with previous studies in sub-Saharan African countries, including Cameroon, Bangladesh and Ghana [51–54], our findings showed that women’s educational attainment and household wealth quintiles, as well as being employed, have significant linear and direct relationships with the use of SBA. This could be attributed to the fact that an increase in a woman’s educational attainment might come with better employment opportunities and an increased household wealth quintile. No doubt, education helps in sensitising women to make good decisions on sexual and reproductive healthcare as well as utilising quality healthcare services provided at various healthcare facilities with their buoyant financial power [50].

As earlier reported in previous studies in Nigeria [35, 55], Cameroon [52], Bangladesh [53], Ghana [54, 56] and Ethiopia [13], our findings further revealed that rural women were significantly disadvantaged in the use of SBA. This is expected given that modern healthcare facilities are mostly located far away from rural residents compared to their urban counterparts, as well as the existing inequality in educational attainment and household wealth quintiles [57]. For the influence of health insurance on maternal healthcare services utilisation, the findings of this study are consistent with studies in Ghana, Rwanda and Tanzania [58–60] that women being covered by health insurance increased their likelihood of using SBA. This has some policy implications because the aim of establishing the National Health Insurance Scheme which is to achieve universal health coverage by the Federal Government of Nigeria in 2004 [61], is fraught with low coverage as observed in this study and a previous study in Nigeria [62]. Hence, the findings of this study plausibly explain the importance of universal access to health insurance schemes among women towards using SBA, especially when confronted with IPV.

Concerning decision-making and the use of SBA, our findings showed that women having decision-making autonomy on their healthcare increased the likelihood of using SBA across the survey years which corroborates previous studies in Burundi and Northern Uganda [63] and Ghana [64]. Surprisingly, in line with previous studies in selected sub-Saharan African countries [2, 65, 66] and Nepal [67], our findings further showed that the likelihood of using SBA significantly was reduced among women who reported making independent decisions on how their earnings are spent in 2008 and 2013. This could be attributed to the recorded largest proportion of women without formal education compared with their counterparts in other categories in 2008 and 2013. Our findings further explain that notwithstanding women having financial autonomy over their earnings, the experience of IPV could generate a state of confusion, shame and powerlessness which may negatively affect their utilisation of skilled maternal healthcare services [7]. Furthermore, the plausible explanation for the findings could be that women who experience IPV might be constrained to compromise seeking skilled healthcare services which provide an opportunity for them to financially take care of some urgent family issues, especially to win the husband’s affection to avoid further abuse and neglect by partners.

### Strengths and limitations of the study

The strengths of the study include the use of a national representative sample drawn from the three consecutive NDHS reports which are useful for the generalisation of the trends in the forms of IPV and SBA utilisation in Nigeria. Also, the study adopted rigorous analytical procedures with weighted proportions. This study has some limitations which include the use of cross-sectional and secondary data from NDHS where IPV measures were taken after the incidence. As a result, the trends in IPV and SBA, as well as the different forms of IPV predicting SBA are relatively temporary and measurement of trends of causal relationships was not easy to ascertain. Also, there is the likelihood of underreporting the experience of IPV by the women who were selected and interviewed for the questions on the domestic violence module during the surveys. The use of life experience of IPV might influence the association since the data were collected for the most recent SBA utilisation. Despite these limitations, the findings are important for more pragmatic policies and programmatic areas of intervention towards reducing IPV prevalence and achieving universal access to skilled maternal healthcare services, especially for disadvantaged women in Nigeria.

## Conclusion

This study demonstrates that despite several efforts and interventions made by the Nigerian government and non-governmental organisations against domestic violence, IPV persists. The prevalence of emotional and physical IPV increased in 2018 after a decrease between 2008 and 2013. There is a gradual increase in sexual IPV and an upward increase in the use of SBA across the survey years. The study further provided evidence that emotional and sexual IPV, unlike physical IPV is associated with low chances of using SBA. Therefore, this study suggests the need to re-strengthen and re-enforce policies and laws on violence against women by their partners which are often accompanied by adverse health outcomes. There should be more pragmatic intervention programmes that focus on specific equity gaps that relate to health insurance coverage and the socio-economic empowerment of women through maternal education, especially the disadvantaged rural dwellers. Such policies and intervention programmes should be aimed at encouraging the use of skilled birth attendants and reducing the prevalence of IPV. This could be a facilitating factor towards achieving the SDG targets of eliminating all forms of violence against all women, maternal and child mortality and promoting the empowerment of women.  

## Data Availability

All datasets from the surveys used for this study are freely available from the DHS Program archive at https://dhsprogram.com/data/dataset_admin/index.cfm. The datasets can be downloaded for use upon requesting to Measure DHS/ICF International, USA.

## Abbreviations

IPV: Intimate Partner Violence
SBA: Skilled Birth Attendance
aOR: Adjusted Odds Ratio
WHO: World Health Organisation
ANC: Antenatal Care
SDGs: Sustainable Development Goals
DHS: Demographic and Health Surveys
NDHS: Nigeria Demographic and Health Surveys
OR: Odds Ratio
CI: Confidence Intervals
Ref: Reference category
